# Complete mitochondrial genome of the harmful algal bloom species *Thalassiosira nordenskioeldii* (Mediophyceae, Bacillariophyta) from the east China sea

**DOI:** 10.1080/23802359.2021.1911715

**Published:** 2021-04-15

**Authors:** Kuiyan Liu, Shuya Liu, Yang Chen, Feng Liu, Nansheng Chen

**Affiliations:** a CAS Key Laboratory of Marine Ecology and Environmental Sciences, Institute of Oceanology, Chinese Academy of Sciences, Qingdao, China; b Laboratory of Marine Ecology and Environmental Science, Qingdao National Laboratory for Marine Science and Technology, Qingdao, China; c School of Earth and Planetary, University of Chinese Academy of Sciences, Beijing, China; d Center for Ocean Mega-Science, Chinese Academy of Sciences, Qingdao, China; e Department of Molecular Biology and Biochemistry, Simon Fraser University, Burnaby, British Columbia, Canada

**Keywords:** Diatoms, HAB species, mitochondrial genome, *Thalassiosira nordenskioeldii*

## Abstract

*Thalassiosira nordenskioeldii* is a common harmful algal bloom (HAB) species with worldwide distribution. Although barcode sequences of this ecologically important species have been published, no genome data have been published for *T. nordenskioeldii*. In this study, we constructed the complete mitochondrial genome (mtDNA) of a *T. nordenskioeldii* strain isolated from the East China Sea. The *T. nordenskioeldii* mtDNA is circular and has a length of 47,038 bp and a GC content of 30.84%. The mtDNA encodes 69 genes, including 40 protein-coding (PCGs), 27 tRNA and two rRNA genes. Phylogenetic analysis using concatenated amino acid sequences of 31 shared PCGs from 37 diatom mtDNAs revealed that the mtDNA of *T. nordenskioeldii* was fully resolved in a clade with that of *Thalassiosira profunda*. The mtDNA of *T*. *nordenskioeldii* showed high collinearity with those of *T. profunda* and *Skeletonema marinoi* with only minor rearrangements. The completion of *Thalassiosira* mtDNAs will facilitate evolutionary studies on species of the order Thalassiosirales and the class Mediophyceae.

The type species of the genus *Thalassiosira* (Cleve 1873), *Thalassiosira nordenskioeldii* Cleve 1873, is a harmful algal bloom (HAB) species with worldwide distribution (Hoppenrath et al. 2007; Li et al. 2013). Although *T. nordenskioeldii* is a common HAB species that frequently causes HABs, molecular information of this species is still limited. Sequences of only a few common molecular markers of *T. nordenskioeldii* including *cox1*, *rbcL* and 18S rDNA have been published (Eharam, Inagaki, et al. 2000a; Ehara, Watanabe, et al. 2000; Alverson et al. 2007). Notably, a group II intron has been characterized in the *cox1* gene of *T. nordenskioeldii* (Ehara, Watanabe, et al. 2000b). Here, we constructed the first complete mtDNA of *T. nordenskioeldii* using the strain CNS00052 isolated from water samples collected in the East China Sea (33°59.934′N, 121°13.59′E) in April 2019. Its specimen is deposited in the collection of marine algae in KLMEES of IOCAS (Nansheng Chen, chenn@qdio.ac.cn) under the voucher number CNS00052.

The total DNA was extracted using DNAsecure Plant Kit (Tiangen Biotech, Beijing, China). The DNA Library was prepared using NEB Next® Ultra™ DNA Library Prep Kit for Illumina (NEB, USA). After qualification, the library was sequenced using an Illumina NovaSeq 6000 platform (Novogene, Beijing). Sequencing results were assembled using Platanus Allee v2.2.2 (Kajitani et al. 2019), and the target scaffolds were selected using BLASTN v2.10.0. BWA v0.7.17 (Li and Durbin 2010) was used to align and verify the circular sequence of the mtDNA. The complete *T. nordenskioeldii* mtDNA (GenBank accession number: MW387419) was 47,038 bp in length with a GC content of 30.84%. The circular mtDNA encodes 69 genes, including 27 tRNA genes, two rRNA genes and 40 protein-coding genes (PCGs), which include five open reading frames (*orf*s) with unknown functions. Introns have been found to occur frequently in *cox1* of *T. nordenskioeldii* strains, such as the CCMP992 strain (AB038235) isolated from the east coast of USA and the CCMP997 strain from Norway (Ehara, Watanabe, et al. 2000). However, no intron was found in the complete *cox1* of the CNS00052 strain, whose *cox1* was 1,521 bp in length encoding 506 amino acids, suggesting high genomic variations among *T. nordenskioeldii* strains. Comparative analysis of the full-length *cox1* genes of *T. nordenskioeldii* strains indicated that the *cox1* genes (AB038235 and AB037974) reported previously (Ehara, Watanabe, et al. 2000) were incomplete. Of the 40 PCGs, 37 initiated with the ATG start codon, while the other three PCGs started with TTG, GTG and ATT, respectively. The stop codons of the 40 PCGs included TAA (31 of 40) and TAG (9 of 40).

To construct the maximum likelihood (ML) phylogenetic tree, all of the 31 PCGs, including *atp6*, *8*, *9*; *cob*; *cox1*, *2*, *3*; *nad1*-*7*, *4 L*, *9*, *11*; *rpl2*, *5*, *6*, *14*, *16*; *rps3*, *4*, *8*, *10*, *11*, *13*, *14*, *19*; and *tatC* from 37 diatom mtDNAs including the mtDNA of *T. nordenskioeldii*, and the mtDNAs of two outgroup taxa, Oomycota species *Phytophthora ramorum* (EU427470) and *Saprolegnia ferax* (NC_005984), were first aligned using MAFFT v7.471 (Katoh and Standley 2013) and trimmed using trimAL v1.4 (Capella-Gutierrez et al. 2009) individually with default parameters (Figure 1). Amino acid sequences of all 31 PCGs of 39 species (including *T. nordenskioeldii*) were concatenated using Phyutility (Smith and Dunn 2008), and the best-fit models for all PCGs were predicted using ModelFinder (Kalyaanamoorthy et al. 2017) with four categories for each partition and a bootstrap value of 1000. The phylogenetic tree was constructed using IQtree v1.6.12 (Trifinopoulos et al. 2016). In the phylogenetic tree, *Thalassiosira nordenskioeldii* grouped with species from *Thalassiosira* and *Skeletonema* species with 100% support, which belong to the class Mediophyceae, order Thalassiosirales (Stoermer 2003). *Thalassiosira nordenskioeldii* clustered well with *T. profunda* (MW013551). The tree topologies was similar to those reported previously by Kaczmarska et al. (2006) and Alverson et al. (2007), which were constructed using the full-length 18S rDNA, the partial 28S rDNA and two chloroplast DNA (*psbC* and *rbcL*) sequences. All phylogenetic trees showed that *T. nordenskioeldii* and *T. profunda* of the family Thalassiosiraceae had closer evolutionary relationship with *Skeletonema marinoi* of the family Skeletonemataceae than that with *T. pseudonana*, although Thalassiosiraceae and Skeletonemataceae were different families in the order Thalassiosirales (Stoermer 2003), supporting the proposal that the taxonomical position of *T. pseudonana* needs to be revised (Alverson et al. 2011). Furthermore, the mtDNA of *T*. *nordenskioeldii* showed high collinearity with those of *T. profunda* and *S. marinoi* (NC_028615) with only minor rearrangements. Two translocation events situated *atp6* and *rps10* to the region between *rns* and *rnl*, and another translocation event brought *atp9* to the position between *nad2* and *nad6*. The completion of more mtDNAs of *Thalassiosira* species is critical for comparing and classifying species of the order Thalassiosirales and the class Mediophyceae.

**Figure 1.**
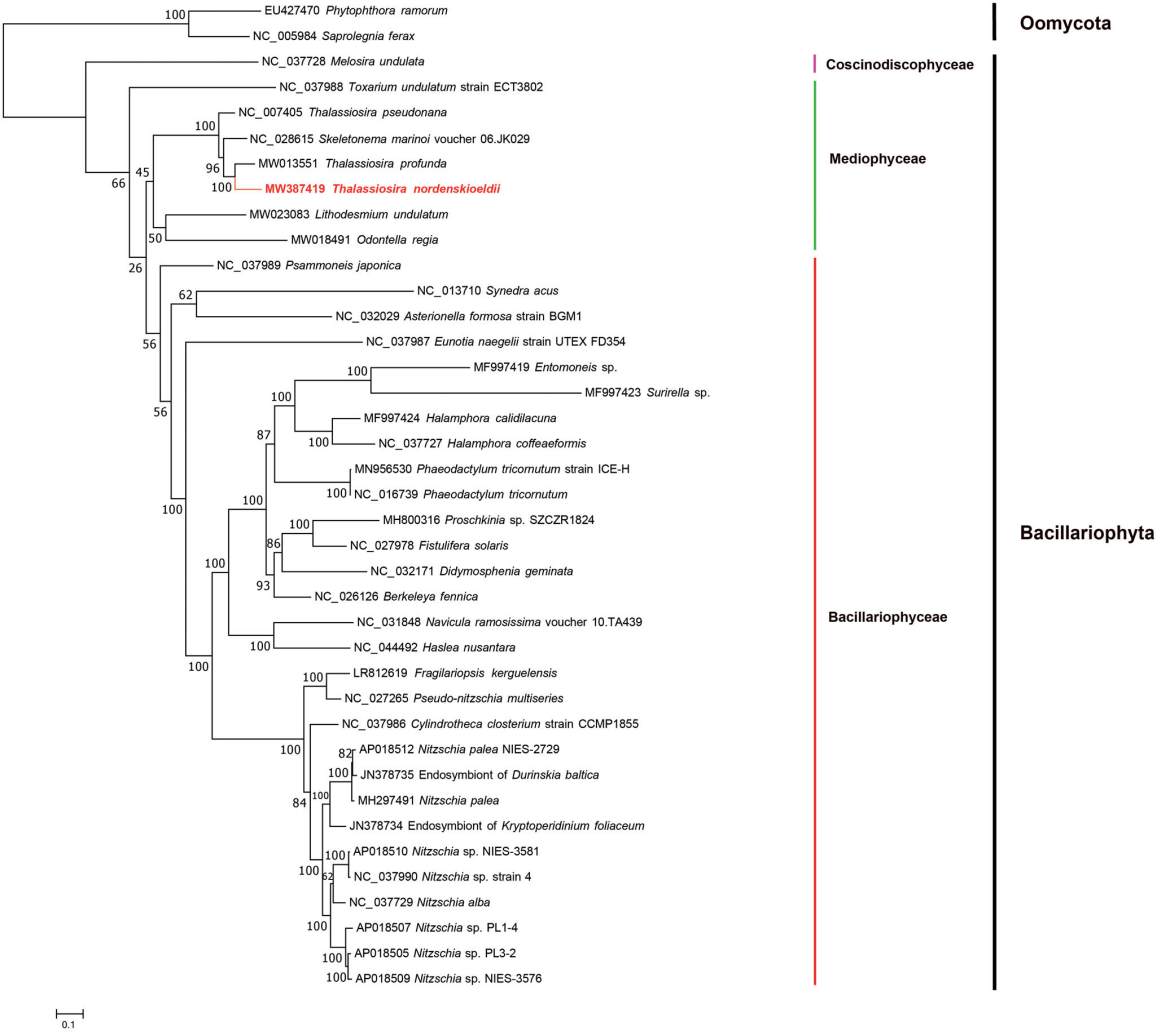
Maximum likelihood (ML) phylogenetic tree of *T. nordenskioeldii* based on concatenated amino acid sequences of 31 genes from 36 diatom mtDNAs, with *Phytophthora ramorum* (EU427470) and *Saprolegnia ferax* (NC_005984) serving as outgroup taxa. The numbers beside branch nodes are the percentages based on 1000 bootstrap replicates.

## Data Availability

The genome sequence data that support the findings of this study are openly available in GenBank of NCBI at https://www.ncbi.nlm.nih.gov/nuccore/MW387419, under the accession no. MW387419. The associated BioProject, SRA and Bio-Sample numbers are PRJNA684688 (https://www.ncbi.nlm.nih.gov/bioproject/PRJNA684688), SRR13279234 (https://www.ncbi.nlm.nih.gov/sra/SRR13279234) and SAMN17125947 (https://www.ncbi.nlm.nih.gov/biosample/SAMN17125947/), respectively.

## References

[CIT0001] Alverson AJ, Beszteri B, Julius ML, Theriot EC. 2011. The model marine diatom Thalassiosira pseudonana likely descended from a freshwater ancestor in the genus Cyclotella. BMC Evol Biol. 11:125–125. 21569560 10.1186/1471-2148-11-125 PMC3121624

[CIT0002] Alverson AJ, Jansen RK, Theriot EC. 2007. Bridging the Rubicon: phylogenetic analysis reveals repeated colonizations of marine and fresh waters by thalassiosiroid diatoms. Mol Phylogenet Evol. 45(1):193–210. 17553708 10.1016/j.ympev.2007.03.024

[CIT0003] Capella-Gutierrez S, Silla-Martinez JM, Gabaldon T. 2009. trimAl: a tool for automated alignment trimming in large-scale phylogenetic analyses. Bioinformatics. 25(15):1972–1973. 19505945 10.1093/bioinformatics/btp348 PMC2712344

[CIT0004] Cleve PT. 1873. On diatoms from the Arctic Sea. Bihang till Kongliga Svenska Vetenskaps-Akademiens Handlingar. 1:1–28.

[CIT0005] Ehara M, Inagaki Y, Watanabe KI, Ohama T. 2000a. Phylogenetic analysis of diatom coxI genes and implications of a fluctuating GC content on mitochondrial genetic code evolution. Curr Genet. 37(1):29–33. 10672441 10.1007/s002940050004

[CIT0006] Ehara M, Watanabe KI, Ohama T. 2000b. Distribution of cognates of group II introns detected in mitochondrial cox1 genes of a diatom and a haptophyte. Gene. 256(1–2):157–167. 11054545 10.1016/s0378-1119(00)00359-0

[CIT0007] Hoppenrath M, Beszteri B, Drebes G, Halliger H, Van Beusekom JEE, Janisch S, Wiltshire KH. 2007. Thalassiosiraspecies (Bacillariophyceae, Thalassiosirales) in the North Sea at Helgoland (German Bight) and Sylt (North Frisian Wadden Sea) – a first approach to assessing diversity. Eur J Phycol. 42(3):271–288.

[CIT0008] Kaczmarska I, Beaton M, Benoit AC, Medlin LK. 2006. Molecular phylogeny of selected members of the order Thalassiosirales (Bacillariophyta) and evolution of the Fultoportula. J Phycol. 42(1):121–138. 10.1111/j.1529-8817.2008.00522.x 27041440

[CIT0009] Kajitani R, Yoshimura D, Okuno M, Minakuchi Y, Kagoshima H, Fujiyama A, Kubokawa K, Kohara Y, Toyoda A, Itoh T. 2019. Platanus-allee is a de novo haplotype assembler enabling a comprehensive access to divergent heterozygous regions. Nat Commun. 10(1):1702. 30979905 10.1038/s41467-019-09575-2 PMC6461651

[CIT0010] Kalyaanamoorthy S, Minh BQ, Wong TKF, von Haeseler A, Jermiin LS. 2017. ModelFinder: fast model selection for accurate phylogenetic estimates. Nat Methods. 14(6):587–589. 28481363 10.1038/nmeth.4285 PMC5453245

[CIT0011] Katoh K, Standley DM. 2013. MAFFT multiple sequence alignment software version 7: improvements in performance and usability. Mol Biol Evol. 30(4):772–780. 23329690 10.1093/molbev/mst010 PMC3603318

[CIT0012] Li H, Durbin R. 2010. Fast and accurate long-read alignment with Burrows-Wheeler transform. Bioinformatics. 26(5):589–595. 20080505 10.1093/bioinformatics/btp698 PMC2828108

[CIT0013] Li Y, Zhao Q, Lü S. 2013. The genus Thalassiosira off the Guangdong coast, South China Sea. Botanica Marina. 56(1):83–110.

[CIT0014] Smith SA, Dunn CW. 2008. Phyutility: a phyloinformatics tool for trees, alignments and molecular data. Bioinformatics. 24(5):715–716. 18227120 10.1093/bioinformatics/btm619

[CIT0015] Stoermer EF, Julius ML. 2003. Centric Diatoms. In: *Freshwater Algae of North America*. (Wehr, J.D. & Sheath, R.G. Eds), pp. 559-594. San Diego: Academic Press.

[CIT0016] Trifinopoulos J, Nguyen LT, von Haeseler A, Minh BQ. 2016. W-IQ-TREE: a fast online phylogenetic tool for maximum likelihood analysis. Nucleic Acids Res. 44(W1):W232–235. 27084950 10.1093/nar/gkw256 PMC4987875

